# Spectral Domain Optical Coherence Tomography Findings in Carotid Artery Disease

**DOI:** 10.4274/tjo.84565

**Published:** 2017-12-25

**Authors:** Akın Çakır, Eyüp Düzgün, Serkan Demir, Yavuz Çakır, Melih Hamdi Ünal

**Affiliations:** 1 University of Health Sciences, Okmeydanı Training and Research Hospital, Department of Ophthalmology, İstanbul, Turkey; 2 University of Health Sciences, Şişli Hamidiye Etfal Training and Research Hospital, Department of Ophthalmology, İstanbul, Turkey; 3 University of Health Sciences, Haydarpaşa Sultan Abdülhamid Han Training and Research Hospital, Department of Neurology, İstanbul, Turkey; 4 University of Health Sciences, Haydarpaşa Sultan Abdülhamid Han Training and Research Hospital, Department of Ophthalmology, İstanbul, Turkey; 5 Private Physician, İstanbul, Turkey

**Keywords:** Carotid artery disease, optical coherence tomography, retinal nerve fiber layer

## Abstract

**Objectives:**

To evaluate the effect of carotid artery disease on retinal morphology by means of spectral domain optical coherence tomography (SD-OCT).

**Materials and Methods:**

We examined 23 eyes with internal carotid artery (ICA) stenosis and 24 age- and gender-matched healthy eyes as a control group in this prospective, case-control study. Compherensive ophthalmic examination and SD-OCT scan were performed to all the patients. The average RNFL and macular thicknesses (MT) in the nine macular ETDRS areas were the major OCT measurements for our study.

**Results:**

Although all of the average RNFL and MT measurements were lower in the ICA stenosis group, only the total MT and outer ETDRS area (temporal/superior/nasal/inferior outer macula) values were found to be significantly thinner compared to the control group (p=0.004, p=0.009, p<0.001, p=0.002, and p=0.001, respectively).

**Conclusion:**

In addition to our knowledge about the effects of ICA stenosis on the retino-choroidal circulation, we found that OCT measurements may be beneficial in the early detection of ocular damage due to ICA stenosis.

## INTRODUCTION

Carotid artery disease (CAD) is characterized by stenosis or occlusion in the carotid arterial system. The most common cause of obstruction is atherosclerosis of the carotid artery, although inflammatory conditions such as giant cell arteritis, fibromuscular dysplasia, and Behçet’s disease can occasionally be responsible.^1^ According to the degree of involvement, especially when the internal carotid artery (ICA) is affected, this may lead to ipsilateral reduced retinal blood flow and eventually progress to ocular ischemic syndrome (OIS). OIS is a rare condition, but its complications may cause severe visual impairment. Most CAD patients have no ocular symptoms when OIS occurs except transient visual loss (amaurosis fugax). Retinal examination may not reveal additional findings at first. As the retinal ischemia becomes chronic, signs and symptoms (mild to severe vision loss, ocular pain, narrowed retinal arteries, dilated but nontortuous retinal veins, and midperipheral dot-and-blot retinal hemorrhages) become prominent.^2^ Since it is a vision-threatening condition, it is important to prevent progression to OIS.

Before the development of ocular findings associated with ICA stenosis, it is believed that the retina may show morphological changes as a result of hemodynamic reduction of ocular circulation. Spectral domain optical coherence tomography (SD-OCT) enables the acquisition of high-resolution images of the retinal layers and detection of retinal nerve fiber layer (RNFL) changes even in the absence of clinical symptoms.^3^

There are limited reports about CAD and retinal layer changes in the literature.^3^ Therefore, in the current study we aimed to analyze the effect of CAD-induced early changes on the retina and RNFL by means of SD-OCT.

## MATERIALS AND METHODS

**Figure 1:**
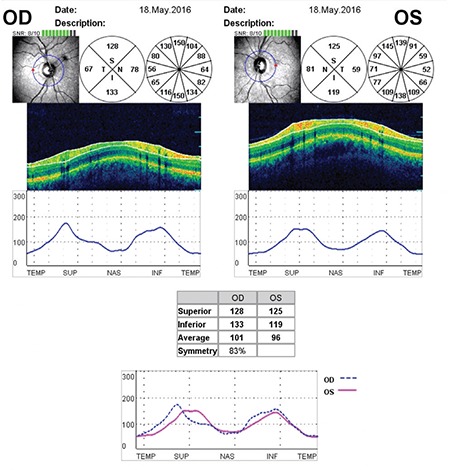
Report of retinal nerve fiber layer thickness measurements OD: Right eye, OS: Left eye

**Figure 2:**
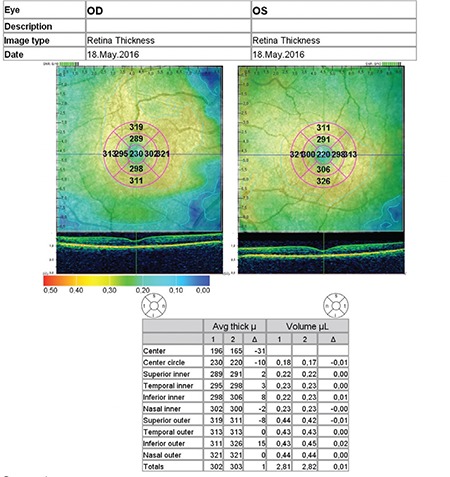
Macular thickness measurement was performed in the nine macular Early Treatment Diabetic Retinopathy Study areas by using the program embedded in spectral domain optical coherence tomography as shown in the figure above OD: Right eye, OS: Left eye

Twenty-three eyes of 23 patients with ICA stenosis greater than 50% (study group) and 24 eyes of 24 age- and gender-matched healthy participants (control group) were involved in this case-control study. The study was approved by the Haydarpaşa Training Hospital Clinical Research Ethical Committee and conducted in accordance with the Declaration of Helsinki. All participants gave written informed consent before enrollment. Exclusion criteria were: ipsilateral external carotid artery (ECA) stenosis, any OIS findings in fundoscopy, any retinal diseases (i.e. glaucoma, diabetes, or retinopathies), history of open or closed-globe injury and vitreoretinal surgery, any neurodegenerative diseases such as Alzheimer and Parkinson’s diseases, any refractive errors greater than 6 diopters, and media opacity that prevented OCT imaging.

articipants who were diagnosed with ICA stenosis using 64-detector-row computed tomography angiography in the Neurology department were referred to our clinic for further investigations. All patients underwent a comprehensive ophthalmic examination including refraction, best-corrected Snellen visual acuity, tonometry, a dilated fundus and slit-lamp examination, and OCT using a Spectral SLO/OCT device (OTI, Toronto, Canada). All the OCT scans were performed by an experienced operator independently and he was masked to the patients’ information. Three continuous RNFL thickness measurements along a circle 3.45 mm in diameter centered at the optic nerve head were obtained and averaged to produce a single RNFL thickness by using the device’s standard program (Figure 1). The average RNFL thickness was taken into consideration in the statistical analysis. Macular thickness (MT) measurement was performed in the nine macular Early Treatment Diabetic Retinopathy Study (ETDRS) areas by using the program embedded in SD-OCT. The ETDRS areas consist of a central 1-mm disc, representing the central MT (CMT), and inner and outer rings of 3 and 6 mm, respectively. The inner and outer rings were divided into four quadrants: superior, nasal, inferior, and temporal (Figure 2).

### Statistical Analysis

SPSS software version 21 (SPSS, Chicago, IL, USA) was used for the statistical analysis. The Shapiro-Wilk test was used to determine whether or not the variables were normally distributed. Student’s t-test was used to compare normally distributed parameters and the Mann-Whitney U test was preferred to compare non-normally distributed variables. When investigating the changes in total MT, the effects of gender and age were adjusted using ANCOVA. For the multivariate analysis, the possible factors identified with univariate analyses were further entered into the linear regression analysis to determine independent predictors of total MT. A 5% type-I error level was used to infer statistical significance.

## RESULTS

Twenty-three eyes of 23 patients, 7 women (30.4%) and 16 men (69.5%), comprised the study group and 24 eyes of 24 healthy individuals, 12 women (50%) and 12 men (50%), comprised the control group in this prospective, case-control study. There was no significant difference between the two groups with respect to age or gender (p=0.095 and p=0.176, respectively). The demographic and clinical features of the study and control groups are summarized in Table 1.

The MT values in each ETDRS quadrant tended to be lower in the eyes with ICA stenosis than controls; however, a statistically significant difference was found only in the total MT and outer ETDRS quadrants (temporal/superior/nasal/inferior outer macula) (p=0.004, p=0.009, p<0.001, p=0.002, and p=0.001, respectively). Likewise, a similar trend was found in the mean RNFL values of the study and control group but there was no statistically significant difference between the groups (97.8±11.07 vs. 103.4±13.2; p=0.120). Table 2 shows the statistical analyses and the mean RNFL and MT values in each ETDRS quadrant of both groups.

Spearman’s correlation analysis showed a mild to moderate, statistically significant negative correlation between the degree of ICA stenosis and total MT, superior outer MT, temporal outer MT, inferior outer MT, and nasal outer MT (Table 3).

In order to eliminate the effect of gender and age on those parameters, we performed ANCOVA. We found that age, gender, and ICA stenosis had statistically significant effect on total MT (p=0.005, p<0.001, and p<0.001, respectively). A multiple linear regression model was used to identify independent predictors. We found that gender and ICA stenosis accounted for most of the effect on total MT (Table 4).

## DISCUSSION

OIS is a rare but vision-threatening condition usually associated with severe carotid artery occlusive disease. The pathogenesis of the syndrome is characterized by decreased arterial inflow on a chronic basis. The duration and degree of the impaired blood flow necessary to develop OIS still is not clear. There is no strict correlation between the degree of CAD and the presence or severity of ipsilateral OIS, probably because there is considerable variation in the capacity of collateral and retrograde filling of the ophthalmic artery from the ECA and the contralateral ICA. Nevertheless, only 5% of the cases progress to OIS due to the presence of collaterals between the ICA and ECA. For instance, while 90% stenosis may not result in OIS in patients with adequate collateral circulation, 50% stenosis may be sufficient to develop OIS in patients with poor collateral circulation.^4^

CAD, especially ICA stenosis, leads to decreased blood flow in the ipsilateral central retinal artery.^3,5^ Although retinal circulation is controlled by local autoregulation, such a prolonged reduction in blood flow may result in some alterations in the retina. Significantly diminished blood flow in the central retinal artery or choroid may cause morphological or functional changes in the retina. Electrophysiological studies show that subclinical abnormalities in patients with carotid artery stenosis precede OIS.^6,7^ Electroretinography has demonstrated that the function of the outer and the middle layers of the retina is suppressed in chronic ocular hypoperfusion a result of reduced oxygen delivery to the eye.^7^

In the current study, we intended to investigate the effect of ICA stenosis on the macular and RNFL thicknesses before the onset of a symptomatic ischemic process. Our results showed that statistically significant thinning occurred in the total macula and outer ETDRS areas before onset of clinical OIS. Although several reports have described changes in ocular blood flow and choroidal thickness in patients with ICA stenosis, there are only two studies in the literature concerning the macular and RNFL thicknesses of the patients with ICA stenosis.^3,8,9,10^

Recently, Sayin et al.^10^ reported that choroidal thickness was lower in patients with ICA stenosis compared with age-matched healthy controls, whereas RNFL, macular, and ganglion cell complex (GCC) thicknesses were similar between the groups. In an another study, Heßler et al.^3^ found no significant differences in RNFL, GCC, or total macular volume parameters between the CAD side and non-CAD side in the entire cohort. However, in this study the authors had to exclude some participants from OCT analysis due to retinal pathologies that could potentially influence measurements, and only 13 patients could be recruited for tests. As far as we know, chronic or intermittent decrease in blood flow to the optic nerve plays an essential role in the pathogenesis of the glaucomatous optic neuropathy.^11^ Likewise, choroidal hypoperfusion results in multiple occlusions of the choriocapillaris and attenuated choroidal vessels.^12^ Therefore, macular and RNFL thinning would not be an unexpected outcome of chronic retinal hypoxia.

Our study is the first to document macular and RNFL thinning in patients with ICA stenosis. However, the results of the current study must be interpreted cautiously. Since this is not a prospective cohort study, many unknown factors may have influenced the results. A multiple linear regression model was performed to determine the independent factors of MTs and interestingly we observed that gender had just as great an influence as ICA stenosis on total MT. A comparable result was reported by Jacobsen et al.,^13^ who found slight but statistically significant effects of average age and gender on retinal thickness asymmetry (0.04 µm/year [0.02-0.06] and 0.54 µm [0.19-0.88 µm], respectively) for men compared with women.^13^ This finding is also supported by a few other studies in the literature.^14,15^ On the other hand, collateral formation between ECA and ICA is also crucial for blood flow regulation of the retina and choroid in CAD. Through these channels, a retrograde flow via the ophthalmic artery to ICA occurs.^16^ Therefore, we excluded the patients with ipsilateral ECA stenosis to achieve a homogenous and reliable assessment.

## CONCLUSION

ICA stenosis leads to a significant reduction in total macular and outer ETDRS area (temporal/superior/nasal/inferior outer macula) thickness prior to the appearance of clinical findings of OIS. These findings may be helpful in the early diagnosis of ocular involvement in patients with CAD.

## Figures and Tables

**Table 1:**
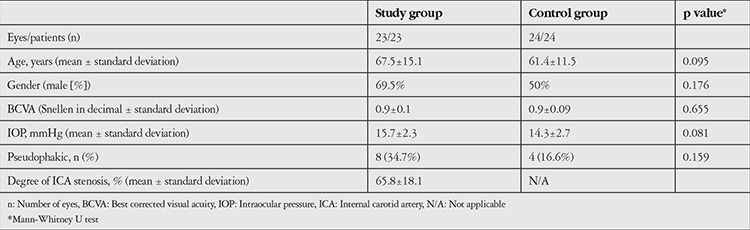
The demographic and clinical features of the study and control groups

**Table 2:**
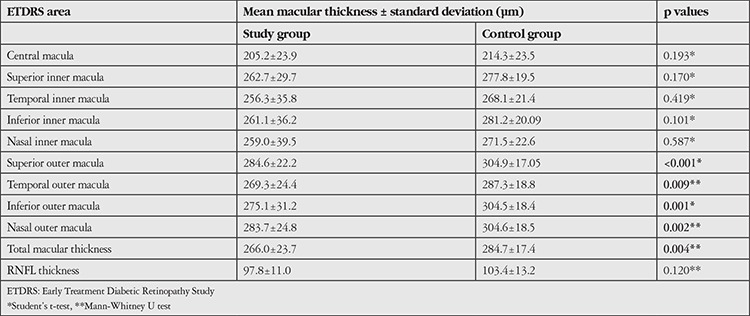
Summary of the mean, retinal nerve fiber layer and macular thickness values in each Early Treatment Diabetic Retinopathy Study areas

**Table 3:**
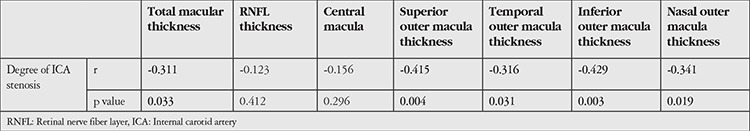
Spearman correlation analysis of the mean retinal nerve fiber layer and macular thickness values in each Early Treatment Diabetic Retinopathy Study area

**Table 4:**
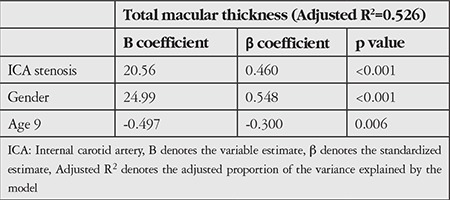
Multiple linear regression modeling for prediction of total macular thickness
